# IIIG9 inhibition in adult ependymal cells changes adherens junctions structure and induces cellular detachment

**DOI:** 10.1038/s41598-021-97948-3

**Published:** 2021-09-17

**Authors:** Victor Baeza, Manuel Cifuentes, Fernando Martínez, Eder Ramírez, Francisco Nualart, Luciano Ferrada, María José Oviedo, Isabelle De Lima, Ninoschka Troncoso, Natalia Saldivia, Katterine Salazar

**Affiliations:** 1 Laboratory of Neurobiology and Stem Cells, NeuroCellT, Department of Cellular Biology, Faculty of Biological Sciences, University of Concepcion, 4030000 Concepcion, Chile; 2 Faculty of Biological Sciences, Center for Advanced Microscopy CMA BIOBIO, University of Concepcion, Concepcion, Chile; 3 Department of Cell Biology, Genetics and Physiology, University of Malaga, IBIMA, Malaga, Spain; 4 Andalusian Center for Nanomedicine and Biotechnology, BIONAND, Malaga, Spain; 5 Networking Research Center on Bioengineering, Biomaterials and Nanomedicine, Malaga, Spain

**Keywords:** Cellular neuroscience, Glial biology

## Abstract

Ependymal cells have multiple apical cilia that line the ventricular surfaces and the central canal of spinal cord. In cancer, the loss of ependymal cell polarity promotes the formation of different types of tumors, such as supratentorial anaplastic ependymomas, which are highly aggressive in children. IIIG9 (PPP1R32) is a protein restricted to adult ependymal cells located in cilia and in the apical cytoplasm and has unknown function. In this work, we studied the expression and localization of IIIG9 in the adherens junctions (cadherin/β-catenin-positive junctions) of adult brain ependymal cells using confocal and transmission electron microscopy. Through in vivo loss-of-function studies, ependymal denudation (single-dose injection experiments of inhibitory adenovirus) was observed, inducing the formation of ependymal cells with a “balloon-like” morphology. These cells had reduced cadherin expression (and/or delocalization) and cleavage of the cell death marker caspase-3, with “cilia rigidity” morphology (probably vibrational beating activity) and ventriculomegaly occurring prior to these events. Finally, after performing continuous infusions of adenovirus for 14 days, we observed total cell denudation and reactive parenchymal astrogliosis. Our data confirmed that IIIG9 is essential for the maintenance of adherens junctions of polarized ependymal cells. Eventually, altered levels of this protein in ependymal cell differentiation may increase ventricular pathologies, such as hydrocephalus or neoplastic transformation.

## Introduction

Adult ependymal cells coexist primarily as E1 multiciliate cells (approximately 50 cilia per cell) and E2 biciliated cells (less than 5% total), lining the walls of the lateral ventricles, the third dorsal ventricle, and the fourth ventricle to create a cerebrospinal fluid (CSF) flow constant^1^. Both cell types constitute highly polarized cells generated from radial glial cells^2–4^, that protect the brain parenchyma from direct contact with CSF, among other functions^5,6^. The stability of this epithelium is maintained by the presence of functional adherens junctions^6,7^. Loss of these junction complexes induces the detachment and death of ependymal cells, generating pathological consequences and changing normal ventricular CSF flow, resulting in ventricular dilation (ventriculomegaly) followed by hydrocephalus and brain tissue edema^8–11^. Additionally, loss of these binding complexes promotes tumorigenic transformation and ependymoma formation in vivo^8,12–15^.

Adherens junctions of the lateral membranes are established by integral membrane proteins and cadherins; their interaction depends on calcium. Intracellular cadherins bind actin microfilaments through a large protein complex that regulates the stability and function of these junctions^16^. Thus, it is relevant to know the proteins and mechanisms that regulate the maintenance of adherens junction in adult ependymal epithelia.

IIIG9, a protein with restricted expression in ciliated tissues, plays a putative role as regulatory subunit 32 of protein phosphatase 1 (PPP1R32) because it contains an RvXF domain in its amino acid sequence^17–19^. In species such as rats and humans, IIIG9 presents two isoforms of different sizes that conserve the RvXF domain, and in the adult brain, the mRNA of this protein is located in ependymal cells that line the ventricular walls, hippocampal neurons, and Purkinje neurons of the cerebellum^20^. Using a specific polyclonal antibody to recognize IIIG9 in rat tissues and through immunohistochemical studies using confocal and electron microscopy, we reported for the first time that IIIG9 presents dotted and discontinuous localization at the edge of the ependymal cilium between the peripheral axoneme and the ciliary membrane^21^. This localization is similar to that described for the PP1 alpha isoform, reported in the cilia of *Chlamydomonas*, where this phosphatase is crucial for the dephosphorylation of the motor protein dynein for flagellar movement and locomotion^22–24^. It is possible that both proteins could be part of a complex that participates in ciliary movement. However, IIIG9 has also been detected in other regions of the cell, suggesting that it may have diverse intracellular functions.

Our present study showed that IIIG9 has intense immunoreactivity in the apical region of ependymal cells, which we have characterized in detail. Additionally, using IIIG9 inhibitor adenoviruses injected into the lateral ventricle, we evaluated the effect of the loss of IIIG9 function in vivo in adult ependymal cells. Taken together, our data indicate that IIIG9 is a key factor for the maintenance of cellular junctions, and its inhibition altered the polarization of ventricular ependymal cells.

## Results

### IIIG9 is detected in adherens junctions of ependymal cells, and its inhibition induces cellular detachment

**Figure 1:**
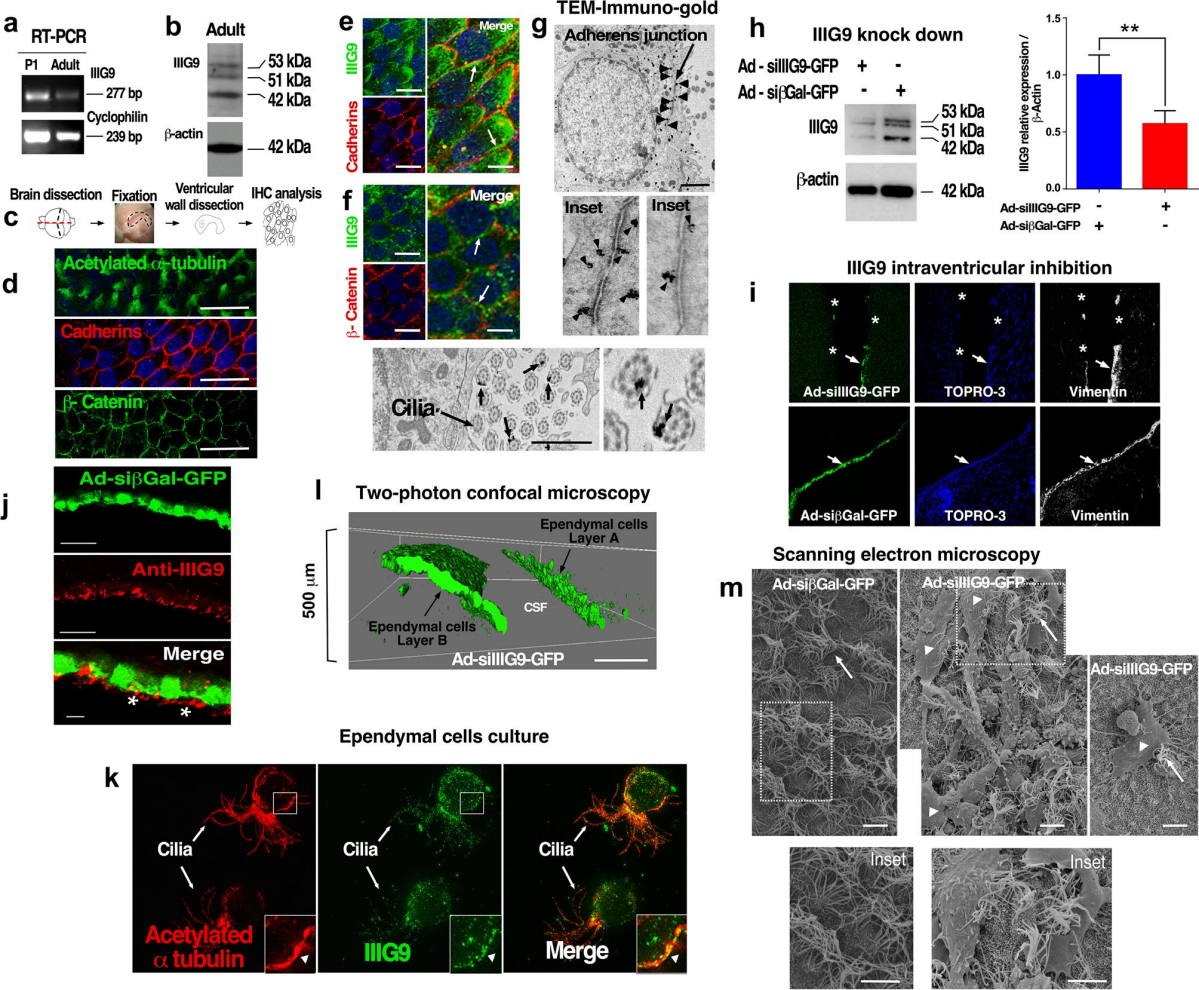
IIIG9 localization in adherens junctions in adult ependymal cells. (**a**) IIIG9 expression analysis using RT-PCR. Samples isolated from postnatal day one (P1) and the adult ventricular wall. (**b**) Western blot analysis. Sample isolated from the adult ventricular wall. Three main bands (42 kDa, 51 kDa and 53 kDa) were detected. (**c**–**f**) *En face* wholemount preparation of the adult lateral ventricular wall and immunohistochemical characterization using anti-acetylated α-tubulin, anti-cadherins and anti-β-catenin antibodies (**d**). IIIG9/cadherin colocalization analysis (**e**, arrows in merged image). IIIG9/β-catenin colocalization analysis (**f**, arrows in merged image). (**g**) *Upper panel*. Immunogold ultrastructural analysis for IIIG9 distribution in ependymal cell adherens junctions (arrowheads and insets). *Lower panel*. Immunogold ultrastructural analysis for IIIG9 distribution in cilia. A positive control immunoreaction. (**h**) Western blot analysis for in vivo IIIG9 knockdown. A representative Western blot is shown for total protein extracts isolated from adult rat ependymal walls after 6 days of i.c.v. injections with Ad-siIIIG9-GFP or Ad-sibGal-GFP. Quantitative analysis of IIIG9 expression by densitometry. (**i**) Upper panel. I.C.V. Ad-siIIIG9-GFP injections (6 days) and vimentin immunodetection. Discontinuous GFP expression (arrows) and negative areas for vimentin (asterisks) were observed in ependymal cells. Lower panel. I.C.V. Ad-siβGal-GFP injections and vimentin immunodetection. Continuous GFP expression (arrows) and a positive reaction for vimentin (arrow) were observed in ependymal cells. Hoechst was used for nuclear staining. (**j**) Ad-siβGal-GFP expression and immunohistochemistry analysis for IIIG9 in the ventricular wall. IIIG9 was located on the cilia (asterisks in merged image) and lateral membranes of the cells. (**k**) Immunohistochemical analysis using anti-IIIG9 and anti-acetylated α-tubulin antibodies in isolated ependymal cells in culture. IIIG9 localization was detected in the cilia and cellular membrane (insets). (**l**) Using 2-photon microscopy, irregular GFP expression in ependymal cells of animals treated with Ad-siIIIG9-GFP was detected (6 days). (**m**) Scanning electron microscopy of the lateral ventricular wall showing ependymal multiciliate cells (arrows and insets) in control animals and a scarce presence of these cells arranged between elongated cells (arrowheads) in Ad-siIIIG9-GFP-injected animals (6 days). All images are representative of different biologically independent samples. CSF, cerebrospinal fluid. (**a**, **b**) n = 3. (**d**–**f**) n = 6. (**g**) n = 2. (**h**) n = 3. (**i**) n = 4. (**j**) n = 3. (**k**) n = 1. (**l**) n = 2. (**m**) = 2. Scale bars = (**d**) 20 μm; (**e**–**f**) 10 μm (lower magnification) and 5 μm (higher magnification); (**g**) 2 μm; (**j**) 40 μm and 10 μm (merge image); (**l**) 50 μm; (**m**) 5 μm. Data is shown t-student (two-tailed) as mean ± SEM. **P < 0.05.

We studied IIIG9 expression in ependymal cells from adult brains (2-month-old rats) by RT-PCR and Western blot analysis. Postnatal day 1 (P1) and adult ventricular wall samples were positive for IIIG9 by RT-PCR analysis, and three principal bands (42, 51 and 53 kDa) were detected by Western blot (Fig. 1a, b; Supplementary Figs. 1, 2) in a similar way to protein extract isolated from trachea (Supplementary Fig. 4). The ventricular zone *en face* wholemount technique is an adequate approach to study the polarity of protein distribution in different cells^25^; thus, we analyzed adult ependymal cells (Fig. 1c–l) using anti-acetylated α-tubulin, anti-β-catenin or anti-cadherin antibodies with this approach (Fig. 1d). Acetylated α-tubulin showed the distinctive conical ciliary projection present in ependymal cells, and detection of cadherins and β-catenin revealed the cell membrane silhouette, with honeycomb characteristics. Additionally, we detected partial colocalization between IIIG9 and cadherins (Fig. 1e, arrows) and between IIIG9 and β-catenin (Fig. 1f, arrows). Furthermore, ultrastructural immunogold analysis showed that IIIG9 was located in close proximity to the adherens junctions (Fig. 1g, upper panel and arrowheads), with a normal distribution in the apical region of polarized ependymal cells. With higher magnification (insets), two types of positive signals were observed, one close to the adherens junctions and another slightly distant from the junctions. Finally, normal IIIG9 localization was confirmed in the ciliary structure (Fig. 1g, lower panel and arrows). The positive signal was polarized to one side of the cilia (gold particles between the membrane and axoneme). Due to its discontinuous distribution, positive cilia alternated with negative cilia for IIIG9 (Fig. 1g, lower panel, arrow and inset).

Using adenovirus to knockdown IIIG9 expression (Fig. 1h, Supplementary Fig. 3), we evaluated the effect in ependymal cells in vivo after I.C.V. injection (Fig. 1i, j, l, m). In the brain, loss of IIIG9 function was studied 6 days after Ad-siβGal-GFP (Ad-Control) or Ad-siIIIG9-GFP (Ad-Knockdown) injection (Fig. 1i), which showed transduction of the ependymal cell layer only; no labeling was detected in the parenchymal area under ependymal cells (Fig. 1i). Using anti-IIIG9 antibodies, we confirmed that Ad-siβGal-GFP did not alter normal IIIG9 expression or distribution (Fig. 1j, red channel). Positive labeling showed an intense immunoreaction in ependymal cilia, but the reaction was also positive intracellularly in lateral cellular membranes (Fig. 1j, asterisk). Similar results were obtained using isolated ependymal cells from the lateral ventricles, with intense IIIG9 immunoreaction observed in cilia and cellular membranes (Fig. 1k, inset and arrows). In Ad-siIIIG9-GFP-injected animals, we observed vimentin/GFP-positive and vimentin/GFP-negative cell regions (Fig. 1i, upper panel, arrows and asterisk). However, in control animals (treated with Ad-siβGal-GFP), only a continuous ependymal layer with vimentin/GFP-positive cells was detected (Fig. 1i, lower panel, arrow). Using 2-photon microscopy analysis to study the ependymal wall by deep imaging, we observed the normal structure of the ependymal layer in Ad-siβGal-GFP-injected animals (data not shown). We also observed ependymal cells without their normal structure on the ependymal layer (Fig. 1l, layer A) following transduction with Ad-siIIIG9-GFP. However, neighboring regions, also transduced by the virus, still showed epithelial integrity (Fig. 1l, layer B), indicating that ependymal cells are differentially affected by the virus. This condition was also observed using SEM, showing cellular debris and cells with elongated processes that intermingled between a few multiciliate ependymal cells (Fig. 1m, arrowheads and arrows). The ependymal cells of control animals confirmed the normal cell structure (Fig. 1m and inset).

**Figure 2:**
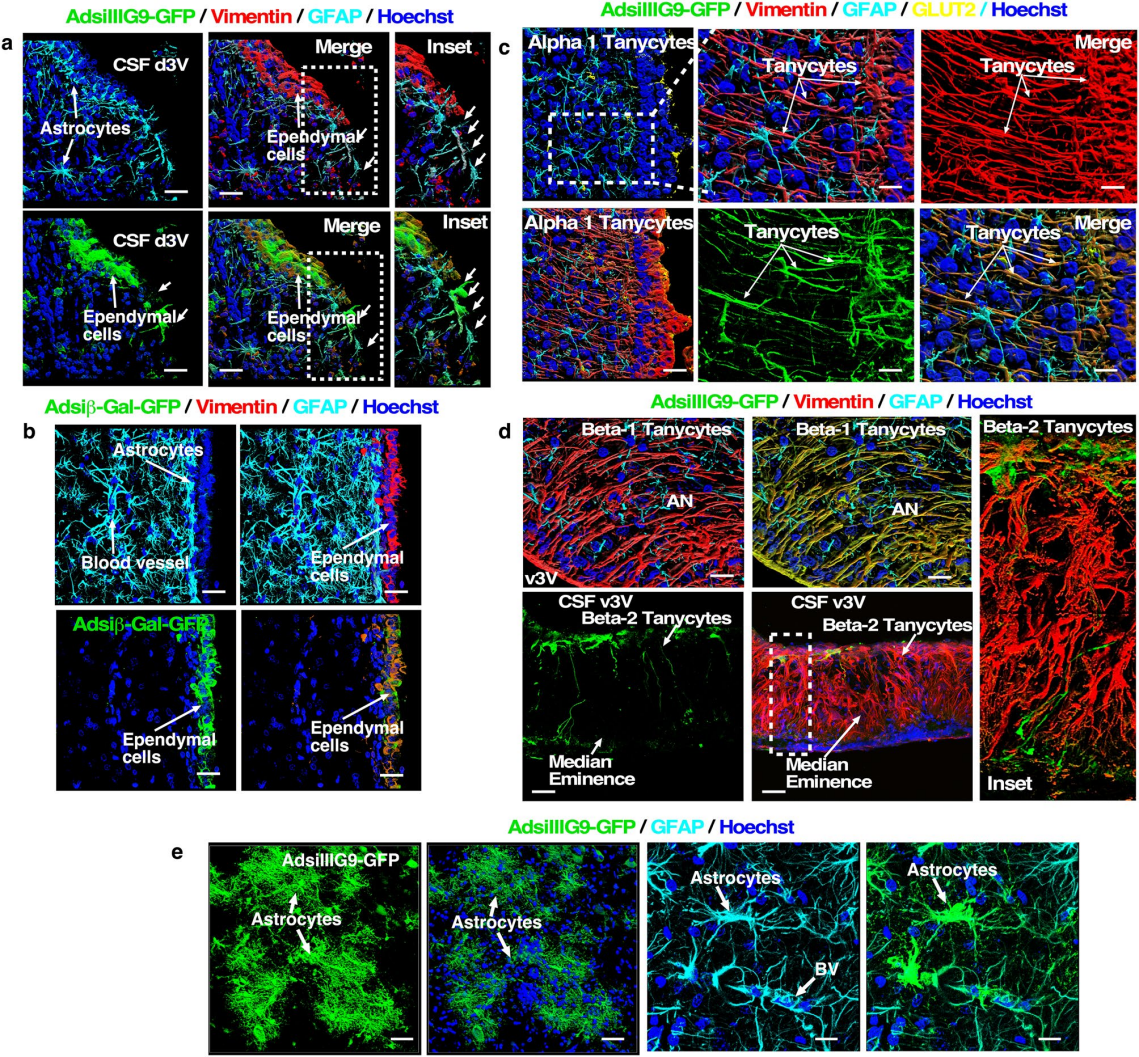
Loss of IIIG9 function in third ventricle ependymal cells induces epithelial denudation but does not alter tanycyte or astrocyte structure. (**a**) Ad-siIIIG9-GFP expression and immunohistochemical analysis for vimentin (red channel) and GFAP (blue-light channel) detection in third ventricle ependymal cells 6 days after Ad-siIIIG9-GFP injection. Confocal Z-stack images were rendered using ZEN software. Ependymal cell denudation was observed (small arrows and insets). (**b**) Ad-siβGal-GFP expression (control) and immunohistochemical analysis for vimentin (red channel) and GFAP (blue-light channel) detection. Ependymal cell denudation was not observed. (**c**) Ad-siIIIG9-GFP expression and immunohistochemistry for vimentin (red channel), GFAP (blue-light channel) and GLUT2 (yellow channel). Tanycytes that were Ad-siIIIG9-GFP/vimentin-positive (brown signal) without structural changes were detected. A normal interrelationship between tanycytes and astrocytes was observed (inset and merged image). (**d**) Ad-siIIIG9-GFP expression and immunohistochemical analysis for vimentin (red channel) and GFAP (blue-light channel) detection. Beta-1 tanycytes that were Ad-siIIIG9-GFP/vimentin-positive (brown signal) without structural changes were detected (upper panels). Ad-siIIIG9-GFP/vimentin-positive cells in the median eminence (green and red channels). Normal cell processes of β2-tanycytes were observed (lower panels and inset). (**e**) Ad-siIIIG9-GFP expression and immunohistochemical analysis for GFAP (blue-light channel) detection in astrocytes from brain tissue. No structural changes were detected. All images are representative of different biologically independent samples. *AN* arcuate nucleus, *BV* blood vessel, *CSF* cerebrospinal fluid, *v3V* ventral third ventricle, *d3V* dorsal third ventricle. (**a**–**e**) n = 3. Scale bars = (**a**–**d**) 10 μm and (**e**) 15 μm.

Similar results were observed when the IIIG9 inhibitor adenovirus was injected into the third ventricle (Fig. 2) by 3D confocal microscopy and image rendering. Vimentin-positive ependymal cells lost their continuity (Fig. 2a, small arrows) without altering periventricular astrocytes (Fig. 2a, astrocytes in the blue-light channel). When injecting the control adenovirus, the ependymal cells (vimentin +) were not altered (Fig. 2b). In these animals, we observed that the GFP virus reached the tanycytes of the third ventricle. Alpha (dorsal to the hypothalamus) and GLUT2-positive tanycytes (Fig. 2c, yellow channel) were rendered to appreciate the projection of their processes (Fig. 2c, red channel) that are interconnected with astrocytes (Fig. 2c, blue-light channel). GFP-positive α-tanycytes showed no alterations in their classic structure (extremely elongated) (Fig. 2c, red channel and merged image—brown signal). In the deepest region of the ventricle (arcuate nucleus), β1-tanycytes positive for AdsiIIIG9-GFP and vimentin (Fig. 2d, brown and red channels, respectively) had normal cellular structure, forming an arc between the third ventricle and the arcuate nucleus neurons. In the median eminence, the viral infection was very low, with only a few cells (β2-tanycytes) positive for AdsiIIIG9-GFP. These positive cells had normal processes, which contacted the external region of the median eminence in some cases (Fig. 2d, lower panel). The β2-tanycyte network was identified with anti-vimentin antibodies (red channel), showing a diffuse network of processes that cross the entire median eminence (inset). Finally, in some animals, adenovirus injection was injected into the periventricular brain parenchyma, where astrocytes showed a positive signal. In these cells, no morphological alterations were observed, and cells with protoplasmic appearance and other astrocytes with fibrous aspects were detected (Fig. 2e, arrows). In addition, the cells were highly positive for GFAP. Thus, AdsiIIIG9-GFP exclusively alters the structure of ependymal cells.

### In vivo IIIG9 inhibition alters the morphology of nonpolarized ependymal cells with lower and delocalized cadherin distribution and increased caspase 3 cleavage

**Figure 3:**
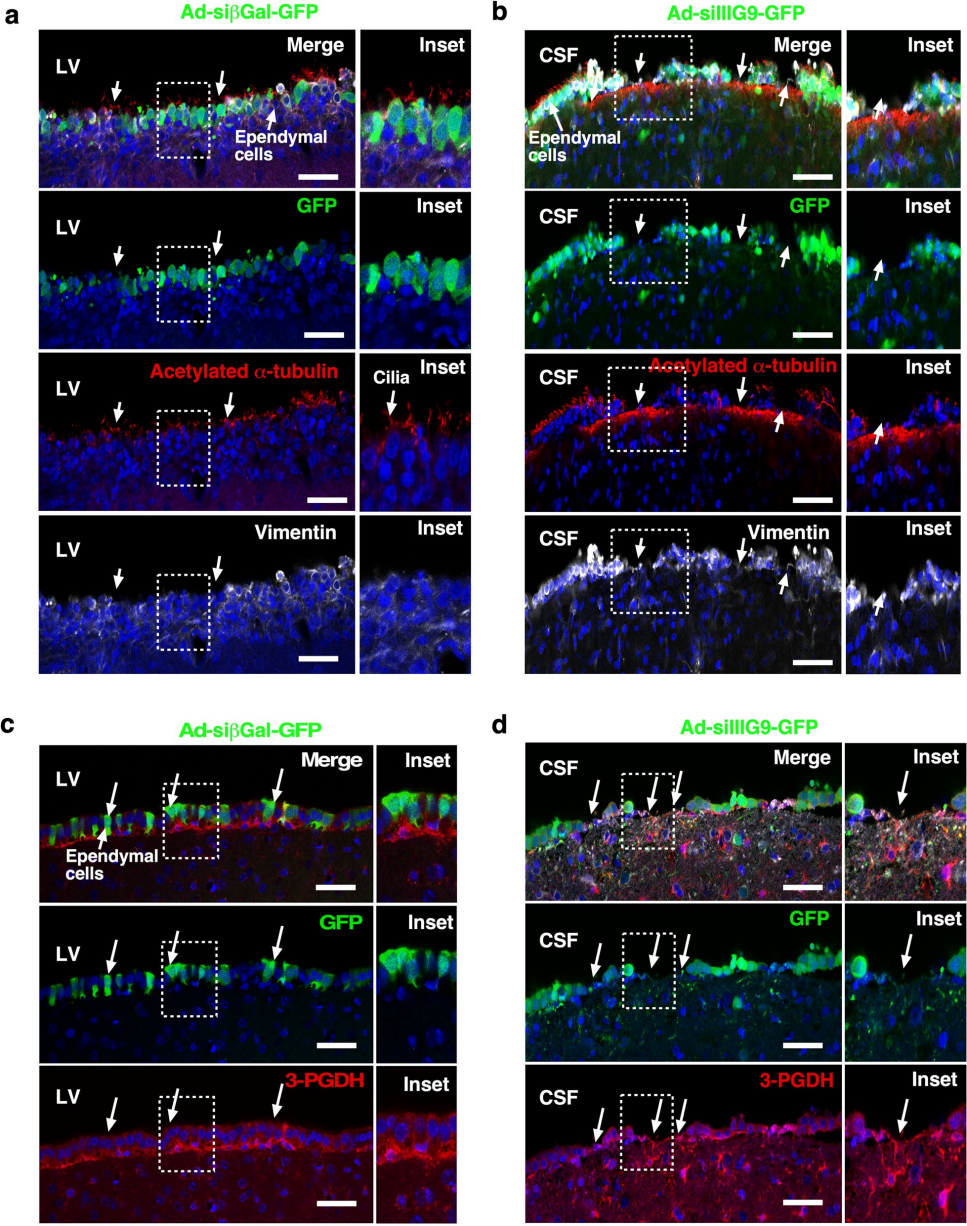
Loss of IIIG9 function in lateral ventricle ependymal cells induces cellular denudation but not astrogliosis. (**a**) Ad-sibGal-GFP expression and immunohistochemistry analysis. Intact ependymal cell layer expressing GFP, acetylated α-tubulin (cilia-red channel) and vimentin (white channel, arrows and insets). The expression of 3-PGDH was also analyzed, and no astrogliosis was detected (**c**, arrows). The 3-PGDH signal was mainly detected in subependymal astrocytes. Scale bar = 40 μm. (**b**) Ad-siIIIG9-GFP expression and immunohistochemical analysis. Irregular ependymal cell layer with expression of GFP, acetylated α-tubulin (cilia-red channel) and vimentin (white channel, arrows and insets). The expression of 3-PGDH was also analyzed, and no astrogliosis was detected (**d**, arrows). 3-PGDH signal was preserved in subependymal astrocytes. All images are representative of different biologically independent samples. *CSF* cerebrospinal fluid, *LV* lateral ventricle. (**a**–**d**) n = 4. Scale bar = 40 μm.

In control animals treated with Ad-siβGal-GFP, GFP-positive ependymal cells showed a normal distribution of acetylated α-tubulin and vimentin (Fig. 3a, arrows and inset). In animals treated with Ad-siIIIG9-GFP for 6 days, we observed narrow denudation areas without GFP expression (Fig. 3b, arrows and inset). In these regions, no positive ependymal cells were observed for acetylated α-tubulin or vimentin. The GFP-positive cells still presented in nearby, nondenudated regions, showing a “balloon-like” shape with vimentin and phosphoglycerate dehydrogenase-3 enzyme (3-PGDH) (Fig. 3c, d, arrows and inset) distributed irregularly inside the cells. 3-PGDH is normally detected in subependymal astrocytes, which do not show hypertrophy. These results indicate that the loss of IIIG9 function (6 days) induces partial ependymal cell denudation without astrocyte activation.

**Figure 4:**
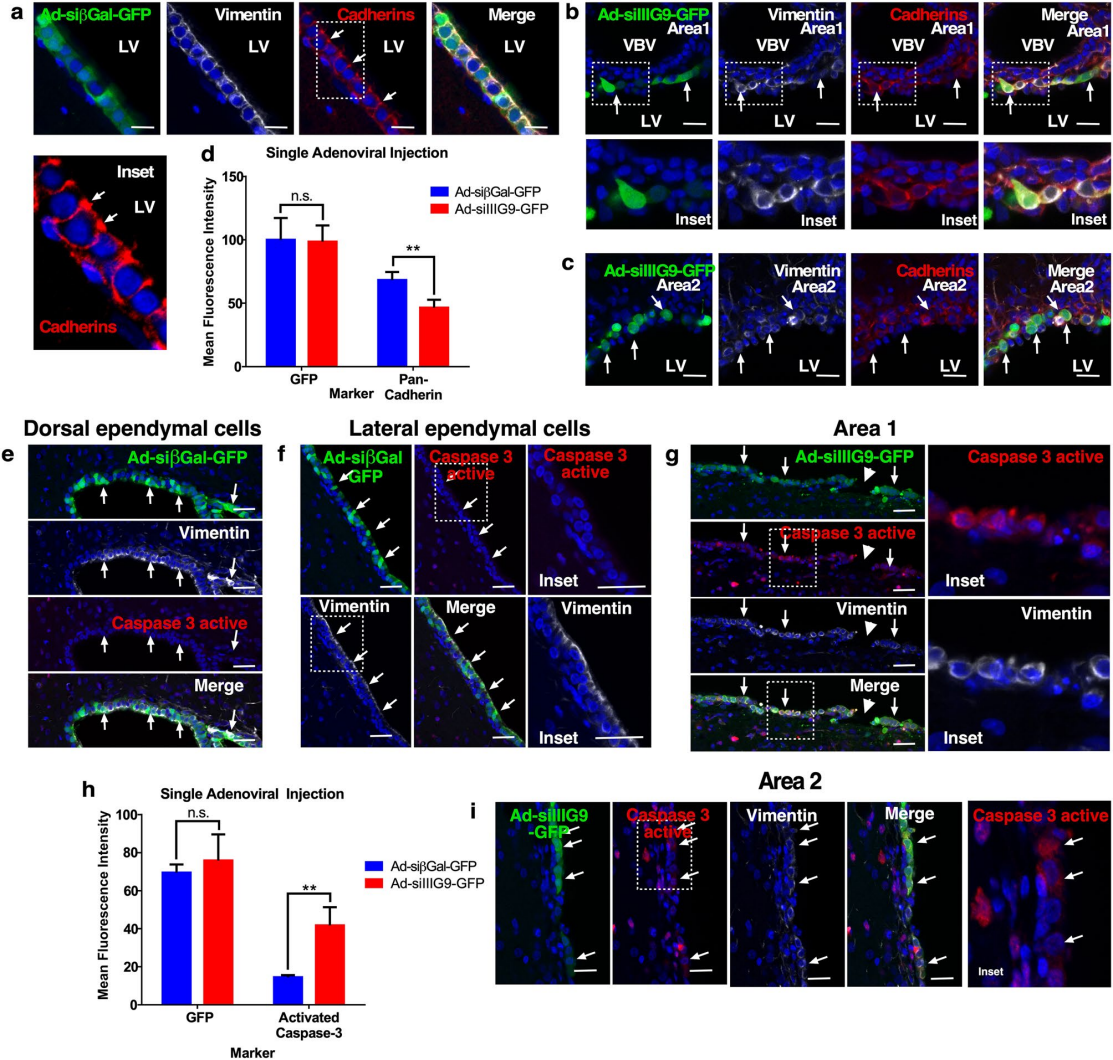
Loss of IIIG9 function induces cadherin redistribution and caspase-3 activation in ependymal cells. (**a**) Ad-siβGal-GFP expression and immunohistochemistry analysis. Ependymal cell layer expressing GFP, vimentin and cadherins (arrows). Cadherins were mainly detected in cell-to-cell contacts (arrows and insets). (**b**) Ad-siIIIG9-GFP expression and immunohistochemistry analysis. Altered ependymal cell layer expressing GFP, vimentin and cadherins (arrows). Lateral ventricle dorsal area. (**c**) Ad-siIIIG9-GFP expression and immunohistochemistry analysis. Altered ependymal cell layer expressing GFP, vimentin and cadherins (arrows). Lateral ventricle lateral area. Reduced cadherin expression (red channel). (**d**) Mean fluorescence intensity plot of decreased cadherin immunoreactivity in ependymal cells from Ad-siIIIG9-GFP animals compared to the controls. Data represent the mean ± SEM from at least four independent biological replicates. (**e**–**f**) Ad-siβGal-GFP expression and immunohistochemistry analysis. GFP, vimentin and caspase-3 active detection in dorsal (**e**) and lateral (**f**) ependymal cells (arrows and insets). (**g**–**i**) Ad-siIIIG9-GFP expression and immunohistochemistry analysis. GFP, vimentin and active caspase-3 detection in the dorsal (**g**) and lateral (**i**) ependymal walls (arrows and insets). Rounded cells with active caspase-3 (arrows and insets) were present near ependymal cell denudation areas (arrowhead and insets). (**h**) Mean fluorescence intensity plot of active caspase-3 increased immunoreactivity in ependymal cells from Ad-siIIIG9-GFP animals compared to the controls. All images are representative of different biologically independent samples. *LV* lateral ventricles, *VBV* blood vessel. (**a**–**c**, **e**–**g**, **i**) n = 4. (**d**, **h**) data are shown t-student (two-tailed) as mean ± SEM; all data are representative of four separate experiments. **P < 0.01, *ns* not significant. Scale bars = (**a**–**c**) 20 μm; (**e**–**f**, **g**, **i**) 40 μm.

Next, we evaluated cadherin localization in control or IIIG9-inhibited animals (Fig. 4a–c). In control animals, cadherins were distributed around the cellular membrane near cell–cell contacts (Fig. 4a, arrows and inset) with normal vimentin distribution (Fig. 4a). In IIIG9-inhibited animals (GFP-positive ependymal cells), we observed mainly intracellular cadherin immunostaining in small and rounded cells (Fig. 4b, arrows and insets). In certain regions of the ependymal wall, the cells showed a very evident “balloon-like” shape (Fig. 4b, c, arrows). Quantitative analysis of the fluorescence intensity for cadherin immunoreactivity showed reduced levels in IIIG9-inhibited animals (46.5 ± 6.2 with respect to 68.2 ± 6.4 in the control, n = 4, **P < 0.01) (Fig. 4d). Finally, analysis of caspase-3 activation showed that it was absent in control ependymal cells (Fig. 4e, f, arrows and insets). Instead, active caspase-3 was intensely detected in small and rounded cells distributed in different areas around ependymal cell denudation (Fig. 4g, i, arrows/arrowheads and insets). Quantitative analysis for active caspase-3 showed an increased signal in ependymal cells from Ad-siIIIG9-GFP animals with respect to the control (41.7 ± 9.6 versus 14.4 ± 1.1 in control, n = 4, **P < 0.01, ns = not significant) (Fig. 4h). Together, our results indicate that the loss of IIIG9 function changes ependymal cell polarization, induces delocalized expression of cadherins and activates caspase-3.

### In vivo IIIG9 inhibition induces moderate ventriculomegaly and ependymal cell cilia stiffness

**Figure 5:**
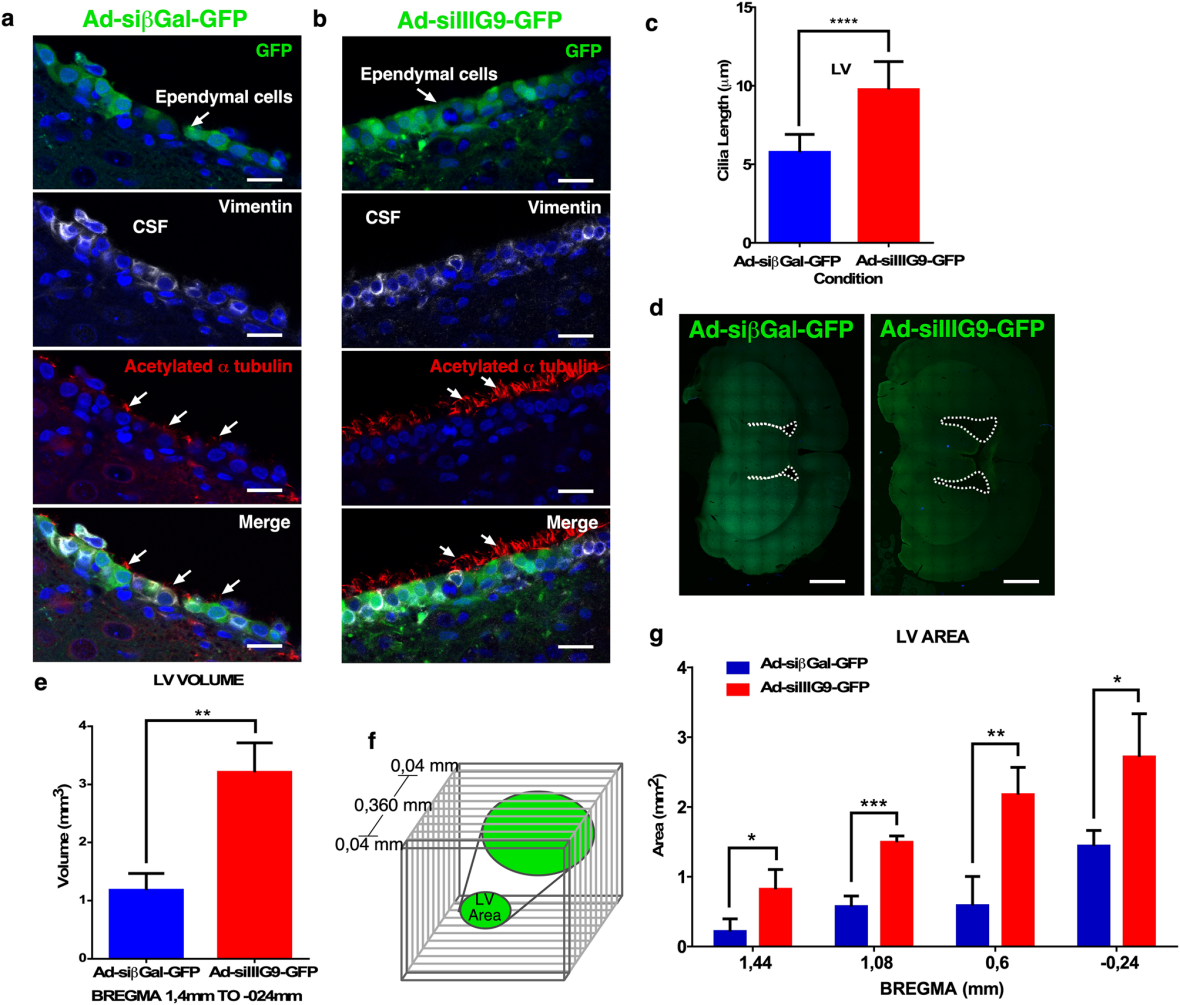
Loss of IIIG9 function in ependymal cells induced greater ciliary length and ventriculomegaly. (**a**) Ad-siβGal-GFP expression and immunohistochemistry analysis. Ependymal cell layer expressing GFP, vimentin and acetylated α-tubulin. Ependymal cells with cilia (arrows), suggesting a reverse stroke position (lying down). (**b**) Ad-siIIIG9-GFP expression and immunohistochemistry analysis. Ependymal cell layer expressing GFP, vimentin and acetylated α-tubulin (arrows). Ependymal cells with cilia (small arrows), suggesting a forward stroke position (straight). (**c**) Quantification analysis of cilia length observed in ependymal cells from the lateral ventricle area in Ad-siIIIG9-GFP-injected animals compared to the controls. (**d**) Tile-scan imaging of brain slices from Ad-sibGal-GFP- or Ad-siIIIG9-GFP-injected animals. Representative dotted lines define the lateral ventricle area observed in control or Ad-siIIIG9-GFP-injected animals. (**e**) Lateral ventricle volume analysis in control or Ad-siIIIG9-GFP-injected animals. (**f**) Schematic representation of the quantification method and the selection of brain sections. (**g**) Lateral ventricular area quantification in different brain bregma positions. All images are representative of different biologically independent samples. *CSF* cerebrospinal fluid. (**a**, **b**) n = 4. (**d**) n = 4. (**c**) Data are shown t-student (two-tailed) as mean ± SEM; all data are representative of four separate experiments. ****P < 0.0001. (**e**–**g**) Data are shown t-student (two-tailed) as mean ± SEM; all data are representative of four separate experiments. *P < 0.05, **P < 0.01, ***P < 0.001. Scale bars = (**a**, **b**) 20 μm; (**d**) 1 mm.

IIIG9 is a protein with ciliary localization in adult ependymal cells, which suggests a role in facilitating PP1 alpha activity in ciliary movement^21^. Immunocytochemical analysis of acetylated α-tubulin (a ciliary marker) showed unusual stiffness, suggesting a ciliary forward stroke position, in the ependymal cells transduced with Ad-siIIIG9-GFP expression (Fig. 5b, arrows), which was not observed in control animals, which showed a reverse stroke position (Fig. 5a, arrows). Quantitative analysis indicated that the cilia length had an average of 5.7 ± 1.1 microns in control animals; however, in the ad-siIIIG9-GFP-injected animals, the average was 9.7 ± 1.7 microns (n = 4, ****P < 0.0001) (Fig. 5c).

Permanent or temporary absence of CSF flow due to ciliary stiffness may affect the ventricular structure; thus, we analyzed whether there were changes in the volume of the lateral ventricle. In animals injected with Ad-siIIIG9-GFP, we detected an enlarged ventricular cavity in contrast to control animals (Fig. 5d, area delimited by the segmented lines). We calculated a ventricular volume of 1.1 microns^3^ in Ad-siIIIG9-GFP-injected animals, which contrasted with the control that had a volume of 3.2 microns^3^ (n = 4, **P < 0.01) (Fig. 5e). Similarly, the area observed in different parts of the ventricle was greater (n = 4, *P < 0.05, **P < 0.01, ***P < 0.001) (Fig. 5f, g). Overall, these results suggest that the early effect of IIIG9 inhibition in ependymal cells is ciliary beating stiffness, which then induces ventriculomegaly in a later step.

### Total ependymal cell denudation and parenchymal astrogliosis are induced after 14 days of IIIG9 inhibition

**Figure 6:**
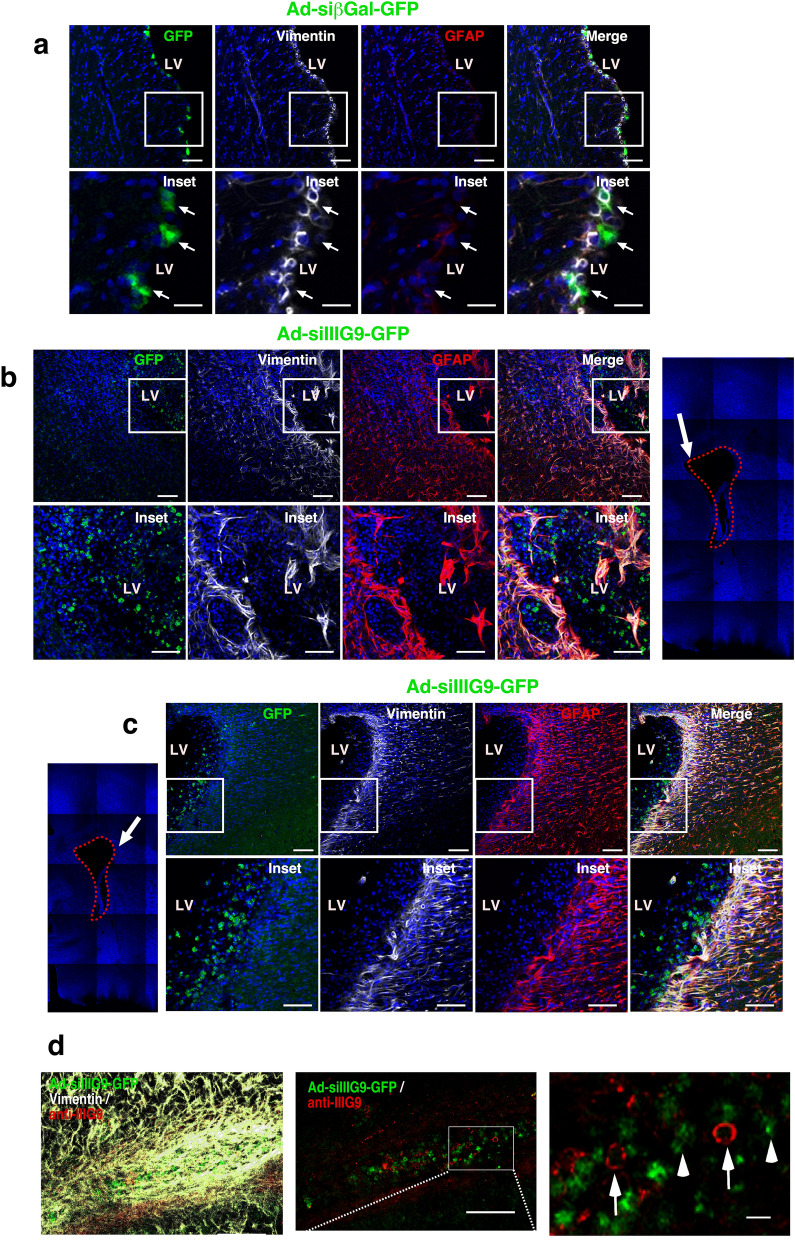
Extended in vivo loss of IIIG9 function induces extensive ependymal denudation and parenchymal reactive gliosis. (**a**) Ad-sibGal-GFP expression and immunohistochemistry analysis. GFP, vimentin and GFAP detection in ventricular ependymal cells from control animals. A continuous ependymal cell layer with intense vimentin expression was detected. (**b**) Ad-siIIIG9-GFP expression and immunohistochemistry analysis. GFP, vimentin and GFAP detection in ventricular ependymal cells 14 days following Ad-siIIIG9-GFP Alzet pump administration. The blue image on the right side indicates the observed area (arrow). A discontinuous ependymal cell layer and astrogliosis (GFAP-positive cells-red channel) were observed (insets). (**c**) Ad-siIIIG9-GFP expression and immunohistochemistry analysis with similar aforementioned analysis (**b**). The blue image on the left side indicates the observed area (arrow), mainly located in the neurogenic niche. Glial cells with positive vimentin processes were mainly detected in this zone. GFAP-positive cells are mainly elongated in form. (**d**) GFP expression and immunohistochemistry analysis for IIIG9 in the ventricular wall from Ad-siIIIG9-GFP-treated animals for 14 days showed that the parenchymal reactive gliosis area was highly positive for vimentin. Some cells were positive for IIIG9 (red channel, long arrows), without colocalization with GFP-positive cells (arrowhead). All images are representative of different biologically independent samples. *LV* lateral ventricles. (**a**–**d**) n = 4. Scales bars = (**a**–**c**) 20 μm; (**d**) 40 μm and 20 μm in the inset.

It has been reported that ependymal denudation (induced by loss of adherens junctions in ependymal cells), is accompanied by parenchymal reactive astrogliosis, which is characterized by astrocyte hypertrophy/hyperplasia with higher expression of intermediate filament proteins, such as vimentin and glial fibrillary acidic protein (GFAP). Microglial cell migration and activation have also been demonstrated. We designed an experimental approach to increase the area of ependymal denudation by continuous infusion of adenoviruses in the lateral ventricle for 14 days using Alzet^®^ osmotic pumps (Fig. 6). In control animals, we observed a continuous ependymal layer with vimentin-positive cells, which were transduced by the control adenovirus, as demonstrated by GFP expression (Fig. 6a, arrows in inset). In addition, we detected discrete GFAP immunoreactivity in subependymal parenchymal astrocytes (Fig. 6a, arrows). In contrast, in Ad-siIIIG9-GFP-treated animals, we observed intense and widely distributed labeling for vimentin and GFAP, where many stellate cells coexpressed both markers (Fig. 6b, arrow in inset). Surprisingly, we detected numerous, rounded GFP-expressing cells inside the ventricular cavity (green channel and inset) that were surrounded by vimentin/GFAP-positive astrocytes (glial scar) (Fig. 6b, merged image and inset). In the neurogenic niche zone (Fig. 6c, arrow in blue image), activated glial cells showed long, vimentin- and GFAP-positive processes. The GFP-positive cells were mainly located inside the ventricle (Fig. 6c, green signal in insets). Finally, IIIG9 was weakly detected in vimentin-positive cells present in astrogliosis induced after Ad-siIIIG9-GFP injection (Fig. 6d, merged image). In thin sections of a similar zone, cells with balloon-like morphology were detected and were positive for GFP but negative for IIIG9 (Fig. 6d, red and green channels and arrowheads in inset). In turn, cells positive for IIIG9 (red signal) were negative for GFP (Fig. 6d, red and green channels and long arrows in inset).

**Figure 7:**
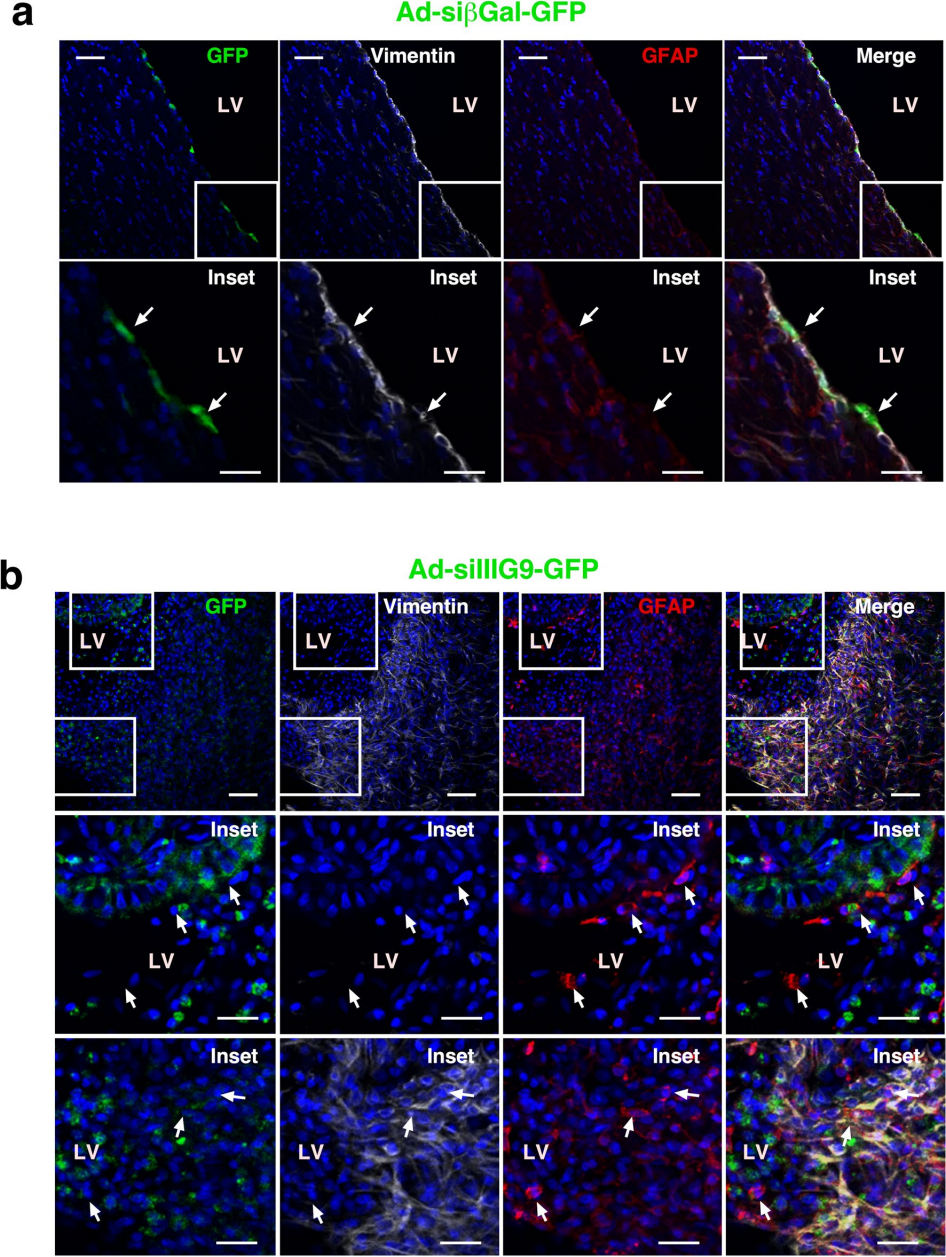
Microglial cell distribution in parenchymal reactive gliosis induced by loss of IIIG9 function. (**a**) Ad-sibGal-GFP expression and immunohistochemistry analysis. GFP, vimentin and IBA-1 detection in ventricular ependymal cells. Diffuse IBA-1 staining was detected below the ependymal cells (arrows in insets). (**b**) Ad-siIIIIG9-GFP expression and immunohistochemistry analysis. GFP, vimentin and IBA-1 were mainly detected in the gliosis area. IBA-1- and GFP-positive cells were also detected in cells inside the ventricular area (inset in upper panel). All images are representative of different biologically independent samples. *LV* lateral ventricle. (**a**, **b**) n = 4. Scale bars = (**a**, **b**) 40 μm.

To analyze the presence of microglia/macrophages in regions with tissue damage, we used the microglial marker IBA-1 (Fig. 7), which was detected in the areas with astrogliosis in Ad-siIIIG9-GFP-treated animals (Fig. 7b, arrows) compared with controls (Fig. 7a, arrows). In control animals, microglia were detected in the subependymal region, but the cells were observed to have fine processes, similar to nonactivated microglia (Fig. 7a, red channel and inset). Conversely, microglia that were detected in the astrogliosis region had thicker processes, showing an activated phenotype (Fig. 7b, red channel and insets). In regions with vimentin-positive cells, the microglial density was even higher, showing a diffuse pattern of IBA1-positive processes (Fig. 7b, red channel and insets-merge). Taken together, our results indicate that in vivo loss of IIIG9 function induces ependymal cell detachment, which is accompanied by parenchymal reactive astrogliosis and possibly IBA1-positive cell migration.

## Discussion

In this work, we have shown that IIIG9 is located in the adherens junctions of ependymal cells of the adult brain. Functional loss of IIIG9 induces ependymal denudation in vivo, where the remaining cells show altered localization and expression of cadherins. These cells also express active caspase 3, indicating that physiological levels of IIIG9 are essential for the maintenance and subsequent survival of polarized ependymal epithelium. This effect, in turn, leads to the appearance of ventriculomegaly associated with the presence of long, straight cilia. Finally, continuous IIIG9 inhibition in the ependymal epithelium induces its total loss and a subsequent parenchymal reactive gliosis response.

Due to the presence of an RVXF domain in IIIG9, it was assigned a putative role as regulatory subunit 32 of PP1. Pull-down assays performed in HEK293T cells using glutathione S-transferase (GST)-tagged PP1alpha have shown that IIIG9 interacts with PP1 in vitro^18^. Incubation of keratinocyte cultures with okadaic acid, a PP1 inhibitor, induces the hyperphosphorylation of β-catenin and loss of cell–cell contacts^26^. Subsequent studies demonstrated that the alpha subunit of PP1 (PP1 alpha) regulates the maintenance of occluding junctions in MDCK cell cultures, where it dephosphorylates serine 824 in partitioning defective 3 homolog protein (PAR-3), inducing the formation of a functional complex between Par-3 and atypic kinase C protein (aPKC) in the apical domain of epithelial cells^27^. Similarly, analysis of the e-cadherin interactome showed that PP1alpha and Par-3 are biological candidates^28^. Furthermore, PP1 alpha has been linked to the positive regulation of the cadherin-catenin complex at cell–cell junctions to facilitate collective migration in a Drosophila border cell migration model^29^. However, in many of these studies, the key protein that focuses on the activity of PP1 at the protein complexes of cell junctions is unknown. In this context, IIIG9 may be a key player in fulfilling this role.

Functional inhibition of cadherins by treatment with specific peptides that bind to the homophilic extracellular domain, histidine-alanine-valine, generates cytological and morphological changes (e.g., nuclear chromatin condensation, scarce cytoplasm, typical round morphology of nonpolarized cells and absence of cilia) in ependymal cells accompanied by the disruption of adherens junctions and the intracellular relocation of cadherins. These changes trigger massive apoptosis and the consequent generalized ependymal denudation of the ventricular walls^8^. The functional loss of IIIG9 in vivo in adult rat brains injected with adenovirus aimed at silencing IIIG9 expression revealed a decrease in the expression and delocalization of cadherins in nonpolarized cells (balloon-like type) present in areas near denudation of the ependymal epithelium. Furthermore, these cells were positive for active caspase 3, a cell death marker, unlike the control animals. Similarly, sustained inhibition studies for 14 days using Alzet pumps showed a more drastic effect, with total ependymal denudation accompanied by a strong parenchymal reactive astrogliosis response and the presence of numerous nonpolarized cells that express the GFP marker of adenoviral inhibition in the ventricular cavity. Reactive astrogliosis was expected because ependymal denudation, which occurs after loss of adherens junctions, is accompanied by a glial scar that isolates neurons from direct contact with CSF^8,11,30–34^. However, the presence of nonpolarized cells in the ventricular cavity (which express the GFP marker of adenoviral transduction) was unexpected. Future studies will help us define the biological identity of these cells. Even though in our study we have used a control adenovirus, we cannot rule out that some of the observed cellular effects are produced by the susceptibility of the ependyma to adenoviral transduction.

In partially denudated animals, ependymal disruption was accompanied by an increase in the volume of the lateral ventricles, a condition known as ventriculomegaly, and a significant increase in the length of the ependymal cilia, as observed by immunostaining for alpha acetylated tubulin. Various in vivo studies on the loss of function of proteins that participate in the movement and/or structure of motile cilia (e.g., knockout animals of FOXJ1, the master transcription factor of ciliogenesis) show the appearance of cerebral ventriculomegaly and hydrocephalus^10,12,35–38^. In tissue microarray studies of cells with motile cilia, such as the airways, fallopian tubes and the brain, co-expression of FOXJ1 and C11orf66 (human IIIG9) was observed^17^, and we have reported that in the rat brain, IIIG9 has a dotted and polarized location towards one edge of the cilia of E1 cells^21^. Thus, we postulated that the loss of IIIG9 first slowed and/or arrested the ciliary beat, with the consequent accumulation of CSF responsible for the ventriculomegaly observed. In mutant mice for the protein Hydin, which is part of the central pair of the ciliary axoneme and is required for flagellar movement, the length and density of the cilia in the ependyma are similar to those of wild-type animals, maintaining the 9 + 2 microtubule conformation, as do the dynein arms and radial spokes, but one of the two central pairs of microtubules loses a specific projection. This causes the cilium of Hydin mutants to beat abnormally, as they are unable to bend normally and simply just vibrate, which is why they appear longer or straighter in phase contrast microscopy. This reduces the frequency of the ciliary beat until ciliary movement stops completely, rendering the ependymal cells unable to maintain adequate flow of the surrounding fluid^39^. Therefore, defects in the central pair of the ciliary axoneme and in the dynein arms impair the dynamics of ciliary movement and the flow of CSF in the brain. Using an *in-house* antibody, we have previously reported that IIIG9 localizes in a dotted pattern between the ciliary axoneme and membrane in adult rat ependymal cells^21^, similar to that detected for the PP1 alpha protein in the cilium of Clamydomonas, where this phosphatase dephosphorylates dynein, facilitating ciliary beating^22^. Similarly, we hypothesize that the absence of IIIG9 initially alters and/or arrests ciliary movement in ependymal cells, which induces an abnormal accumulation of CSF in the lateral ventricles, explaining the ventriculomegaly and the presence of long, straight cilia in a similar fashion as in the Hydin mutant. Finally, the sustained loss of IIIG9 in these cells would alter the stability of cadherins in the adherens junctions, triggering the disassembly of these junctions, the appearance of nonpolarized round cells and a cell death response that would lead to the observed ependymal detachment.

Finally, our study suggests new functions for IIIG9 as an essential protein in the physiology of adult ependyma, and its regulation may be involved in pathologies present in children and adults that present with ependymal denudation, such as hydrocephalus.

## Materials and methods

### Animals

Sprague-Dawley rats 2 months of age were used. The average weight of the adult rats was 275 ± 25 g. Animals were kept in a 12-h light/12-h dark cycle with food and water ad libitum. Animal work, procedures and methods were approved by the Ethics and Animal Care and Use Committee of the University of Concepcion (Chile), Grant Fondecyt 1190848, therefore, all methods were performed in accordance with the relevant guidelines and regulations of the same University. Additionally, the study is reported in accordance with ARRIVE guidelines (https://arriveguidelines.org).

### RT-PCR

Total RNA extracts were isolated from postnatal day 1 (PN1) and adult rats using TRIzol Reagent^®^, and RT-PCR was conducted as previously described^40^. The following primers (based on the rat sequence GenBank accession number AY032665.1) were used to analyze the expression of IIIG9: forward 5′-ACAACCCCAGCTATGTTCGG-3′ and reverse 5′-GGCACGTCTCGATAGAAGGG-3′ (expected product 277 base pairs [bp]). The following sets of primers were used to analyze the expression of the housekeeping gene GAPDH: forward 5′-GCAAGTTCAACGGCACAGTCAAG-3′ and reverse 5′-CGTGGTTCACACCCATCACAAAC-3′ (expected product 350 bp). To verify the results, all experiments were performed in triplicate.

### Western blotting

Total protein extracts were isolated from adult ependymal wall explants or trachea as previously described^40^. Briefly, the samples were obtained by ultrasonic homogenization in ice-cold isotonic buffer with protease inhibitors. Total protein fractions were collected from the supernatant after debris and nuclear sedimentation (10,000 g for 10 min at 4 °C). In total, 10 μg, 20 μg or 100 μg of protein was loaded in each lane, separated by sodium dodecyl sulfate–polyacrylamide gel electrophoresis (gradient 5–15%), transferred to PVDF membranes, and probed with anti-IIIG9 antibodies (1:10,000, in-house rabbit polyclonal antibody), anti-GFP (1:2000, Aves Lab, CA, USA) and anti-b-actin (1:10,000, Santa Cruz, CA, USA). Following incubation with an HRP-conjugated goat anti-rabbit IgG (1:5000; Jackson ImmunoResearch), HRP-conjugated goat anti-mouse IgG (1:5000; Jackson ImmunoResearch) and HRP-conjugated goat anti-chicken IgG (1:5000; Jackson ImmunoResearch) the reaction was developed using the ImageQuant LAS500 enhanced chemiluminescence system (General Electric, Fairfield, CT, USA).

### Immunocytochemistry

Adult rat brains fixed by immersion in 4% paraformaldehyde were used for wholemount lateral ventricle preparation. Additionally, brains injected with adenovirus were dissected and fixed by immersion in 4% paraformaldehyde. Thick frontal sections of the lateral ventricles (40 µm) were cut with a cryostat. The samples were coincubated overnight at 22 °C with the following primary antibodies: anti-IIIG9 antibodies (1:1000, in-house rabbit polyclonal)^21^, anti-vimentin antibodies (#AB5733, Millipore, Temecula, CA, USA, 1:400), anti-3-PGDH antibodies (1:300, kindly donated by Dr. Shigeki Furuya), anti-GFAP antibodies (#Z0334, DAKO, Carpinteria, CA, USA, 1:200), anti-pan-cadherin antibodies (#C1821, Sigma-Aldrich, St. Louis, MO, USA, 1:500), anti-β-catenin antibodies (E-5, #SC-7963, Santa Cruz Biotechnology, Santa Cruz, CA, USA, 1:200), anti-acetylated α tubulin antibodies (#T6793, Sigma-Aldrich, 1:4000), anti-IBA1 antibodies (#AB5076, Abcam, Cambridge, UK, 1:100), anti-cleaved caspase 3 antibodies (#AB3623, Millipore, Temecula, CA, USA, 1:50) and anti-GLUT2 antibodies (Alpha Diagnostic, San Antonio, Tx, USA). The antibodies were diluted in Tris-HCl phosphate buffer (10 mM Tris, 120 mM NaCl, 8.4 mM Na_2_HPO4, 3.5 mM KH_2_PO4, pH 7.8) with 1% bovine serum albumin (BSA) and 0.2% Triton X-100. After extensive washing, the samples were incubated for 2 h with Cy2-conjugated affinity purified donkey anti-rabbit IgG, Cy3-conjugated affinity purified donkey anti-rabbit IgG, Cy3-conjugated affinity-purified donkey anti-mouse IgG, Cy5-conjugated affinity-purified donkey anti-mouse IgG and Cy5-conjugated affinity-purified donkey anti-chicken IgG. All secondary antibodies were used at a 1:200 dilution and were acquired from Jackson ImmunoResearch (West Grove, PA, USA). For nuclear staining, Hoechst 33342 (1:500, Molecular Probes, Eugene, Oregon, USA) was used. In some cases, the images acquired in the confocal microscope using the Z-stack and tiling functions (4 or 5 channels) were rendered using ZEN software 1.0 (blue edition, 2012) (https://www.zeiss.com/microscopy/us/products/microscope-software/zen.html, Zeiss, Germany).

### Transmission/scanning electron microscopy and immunogold staining (TEM–IG)

Brain samples from animals injected with adenovirus were used for scanning electron microscopy (SEM) as previously described^41^. For conventional transmission electron microscopy (TEM), sections (90 or 150 μm) fixed in 2% paraformaldehyde and 0.5% glutaraldehyde were rinsed in 0.1 M phosphate buffer and then postfixed in 2% osmium tetroxide for 1 h. After the sections were rinsed, they were stained with 2% uranyl acetate in 70% ethanol for 3 h, dehydrated in ascending concentrations of alcohol and incubated with propylene oxide for Araldite embedding. Once plasticized, the sections were cured at 60 °C for 3 days. Serial semithin sections (1.5 μm) were cut on an ultramicrotome (Leica, Wetzlar, Germany) and then stained with 1% toluidine blue. Subsequently, ultrathin (60 nm) sections were cut with a diamond knife using the same ultramicrotome and examined under a Jeol Jem-1400 electron microscope. For immunogold electron microscopy (TEM-IG), pre-embedding immunogold staining was performed as previously described^42,43^. Briefly, rats were deeply anesthetized with 250 mg/kg body weight tribromoethanol (Avertin, Chemos GmbH, Regenstauf, Germany) and perfused transcardially with saline (0.9% NaCl), followed by 2% paraformaldehyde/0.5% glutaraldehyde in 0.1 M phosphate buffer. Brains were removed, postfixed by immersion in 2% paraformaldehyde at 4 °C overnight, and cut into 1 μm semithin sections using a vibratome. The sections were first incubated with anti-IIIG9 antibodies diluted 1:1000 (in-house rabbit polyclonal antibody) at 4 °C for 4 days, washed and further incubated with secondary donkey anti-rabbit IgG conjugated to colloidal ultrasmall gold particles (EMS-#25801) at a 1:50 dilution for 24 h at 4 °C. After enhancement of gold particles with silver, sections were washed, postfixed with 2.5% glutaraldehyde for 20 min, washed, and finally postfixed with 1% osmium tetroxide. The antibodies were diluted in blocking buffer (0.5% BSA, 0.1% Fish Gelatin in 0.1 M phosphate buffer, pH 7.4). The tissue sections were dehydrated and embedded in Araldite 502 (EMS). Ultrathin sections (60-nm thickness) were slightly stained with uranyl acetate for analysis with an electron microscope (Philips CM100).

### Primary culture of isolated ependymal cells

Primary cultures were obtained from the septal and striatal ependymal walls from adult Sprague-Dawley rats and maintained in a humidified chamber at 37 °C and 5% CO_2_, as described by Grondona et al.^44^. Briefly, the brain was placed on a glass Petri dish full of cold Mg^2+^/Ca^2+^-free Hanks medium (Invitrogen, Carlsbad, CA, USA). Using a scalpel, brain hemispheres were separated by a transverse cut at the level of the optic chiasm, and then two perpendicular cuts were performed at the dorsal and ventral portions of both lateral ventricles. Therefore, two portions were obtained per hemisphere, corresponding to explants from the septal and striatal ventricular walls. Explants were washed with the same Hanks medium for 30 min at 4 °C and then sequentially incubated with TrypLE™ Express (Invitrogen, Carlsbad, CA, USA) for 5 min at 4 °C, 20 min at 37 °C and 5 min at 4 °C. Next, explants were carefully placed on 10-cm diameter culture dishes and incubated at 37 °C in restrictive medium containing α-MEM supplemented with 0.3% glucose (w/v), 10 mM HEPES, 0.2% Pluronic F127 (w/v) and 0.01% DNAse (w/v). An additional aliquot of 0.01% DNAse (w/v) was added after 6 h. After 24 h, several isolated ependymal cells floated and moved around the medium. To incubate ependymal cells for longer periods, the supernatant was centrifuged at 300 g for 15 min, the pellet was resuspended in DNAse-free restricted medium, and the cells were further incubated in the chamber at 37 °C for 2 DIV (days in vitro).

### Generation of adenoviral vectors

To inhibit IIIG9 expression (GenBank NM_145786.1), we designed adenoviral vectors carrying the siRNA sequences for IIIG9 and β-galactosidase as a control. Using the siRNA design tool (Dharmacon. Inc.), oligonucleotide sequences carrying the shRNA sequences were designed for IIIG9 (si-IIIG9-sn1: 5′-CGCGCCGCTTCATGACTTCCGAGTATTTCAAGAGAATACTCGGAAGTCATGAAGTTTTTTCCTTAAT-3′; si-IIIG9-as1: 5′-TAAGGAAAAAAGTTCATGACTTCCGAGTATTCTCTTGAAATACTCGGAAGTCATGAAGCGG-3′) and β-galactosidase from *E. coli* (si-*E. coli* βgal-sn: 5′-CGCGCCAAGGCCAGACGAGAATTATTTCAAGAGAATAATTCGCGTCTGGCTTTTTTTTTTAAT-3′; si-*E. coli*-βgal-as: 5′- TAAAAAAAAAAGGCCAGACGCGAATTATTCTCTTGAAATATTCGCGTCTGGCCTTGG-3′). Each oligonucleotide pair was aligned and cloned into pDC311.2-OFF-EGFP, which has a multicloning site under the control of the H1 promoter for small RNAs. This vector also encodes the enhanced green fluorescent protein (EGFP) under the control of the human ubiquitin promoter. Adenoviruses were produced by cotransfection of the pBHGloxDE1,3Cre vector with the pDC311.2-IIIG9 or pDC311.2-βgal vectors in HEK293A cells (Invitrogen), which are competent for the production of adenoviral particles. For cotransfection, 10^5^ cells were seeded per well (10 cm^2^) and cotransfected using Lipofectamine 2000 (Invitrogen) at a molar ratio of 1:4. The cells were kept in culture for 10 days with replacement of medium (DMEM plus 5% CFS) every 3 days. Detection of a cytopathic effect and EGFP expression was expected by day 10, when adenoviruses were harvested by freezing (− 70 °C) and thawing (37 °C) three times. This procedure was repeated four times until an adenovirus titer of 10^9^ cfu was obtained. Adenovirus titration was carried out in HEK293T cells (Invitrogen), and the expression of the EGFP reporter gene was observed in serial dilutions.

### In vivo i.c.v. injections of adenovirus in adult animals

Adult rats (2 months) were used to perform single injections (n = 10/condition) and continuous treatments with Alzet^®^ (Alzet O.P. Company) pumps (n = 10/condition). The animals were anesthetized with CO_2_ inhalation, and 20 μL of each respective adenovirus (2 × 10^7^ infectious units for both adenovirus) was injected through stereotaxic approaches (− 0.8 mm, 3.7 DV, 1.5 for the lateral ventricle, and − 3.14 mm, 0 DV, − 9.6 for the third ventricle). The animals were kept for another 6 days. The Alzet osmotic pump was implanted subcutaneously on the back of the animal by a cannula that was attached to the skull following the same stereotaxic coordinates. The pump released 5 μL/h for 14 days. After completion of both experiments, the animals were anesthetized with 2,2,2-tribromoethanol (300 mg/kg i.p.) and fixed with 4% paraformaldehyde by intracardiac perfusion. Additionally, animals were injected with Ad-siIIIG9-GFP (n = 4) or Ad-sibGal-GFP (n = 4) for 6 days after which total protein extracts from ependymal wall explants were isolated and evaluated for IIIG9 knockdown efficiency.

### Quantitative and statistical analyses

Single adenovirus injection images were acquired with the same set of parameters between conditions. Quantification of the mean fluorescence intensity (MFI) was performed using ImageJ software (NIH). GFP + regions of interest (ROIs = 16, in a total of 4 samples of each condition) in the ependymal layer were selected for both control and siIIIG9 adenoviruses to measure the MFI for activated caspase-3 and pan-cadherin staining. Images were acquired with the same set of parameters between conditions. Additionally, in the same samples, the cilia length positive to acetylated a-tubulin were measured manually from control (ROIs = 105) and siIIIG9 (ROIs = 89) in a total of four animals for each condition and using the Profile tool from ZEN software (LSM780 Zeiss confocal microscope). For volume quantification, coronal brain slices (40 μm thick) were mounted in serial distribution (one-each-ten sections), and images were taken on an epifluorescence microscope (Nikon) using a 4 × objective. Slices were ordered by their Bregma coordinates. Quantification of the lateral ventricle area was performed using ImageJ software (NIH). The lateral ventricle volume (from bregma 1.44 mm to − 0.24 mm) was obtained by the total volume slices and the space between them, and the gap volume was calculated by the truncated cone volume formula. The statistical analysis was performed using Prism version 6.01 software (GraphPad Software, San Diego, CA, USA). Data represent the mean ± SD of three or four independent experiments with each determination performed in duplicate. Statistical comparisons between two groups of data were carried out using the unpaired Student’s t-test and analysis of variance (one-way ANOVA, followed by Bonferroni post-test) for multiple comparisons.

## Supplementary Information

Supplementary Figures.

## Data Availability

All data generated or analyzed during this study are included in this published article.
