# Supplementary material for: IIIG9 inhibition in adult ependymal cells changes adherens junctions structure and induces cellular detachment
Source: Sci Rep. 2021 Sep 17;11:18537. doi: 10.1038/s41598-021-97948-3 (PMC8448829; doi:10.1038/s41598-021-97948-3)
Supplement: Supplementary file 1 — Supplementary Figures. [file 41598_2021_97948_MOESM1_ESM.pdf]

Supplementary material

IIIG9 inhibition in adult ependymal cells changes adherens junctions structure and induces cellular detachment

Victor Baeza1#, Manuel Cifuentes3#, Fernando Martínez1, Eder Ramírez1, Francisco Nualart1,2, Luciano Ferrada2, María José Oviedo1, Isabelle De Lima1, Ninoschka Troncoso1, Natalia Saldivia1 and Katterine Salazar1,2*

[1]Laboratory of Neurobiology and Stem Cells, NeuroCellT, Department of Cellular Biology, Faculty of Biological Sciences, University of Concepcion, Concepción, Chile. [2]Center for Advanced Microscopy CMA BIOBIO, Faculty of Biological Sciences, University of Concepcion, Concepción, Chile. [3] Department of Cell Biology, Genetics and Physiology, University of Malaga, IBIMA, BIONAND, Andalusian Center for Nanomedicine and Biotechnology and Networking Research Center on Bioengineering, Biomaterials and Nanomedicine, Malaga, Spain.

*Address correspondence and reprint requests to K. Salazar, Departamento de Biología Celular, Facultad de Ciencias Biológicas, Universidad de Concepción, Barrio Universitario, Concepción 4030000, Chile. E-mail: katterinsalazar@udec.cl

[#] These authors contributed equally

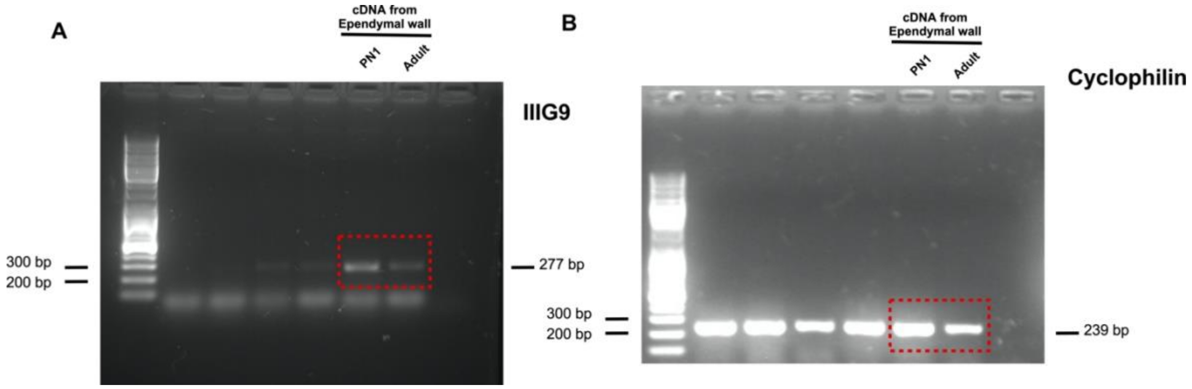

Suppl. Fig. 1. Unedited image gel figure 1a. Red broken lines indicate dropped band.

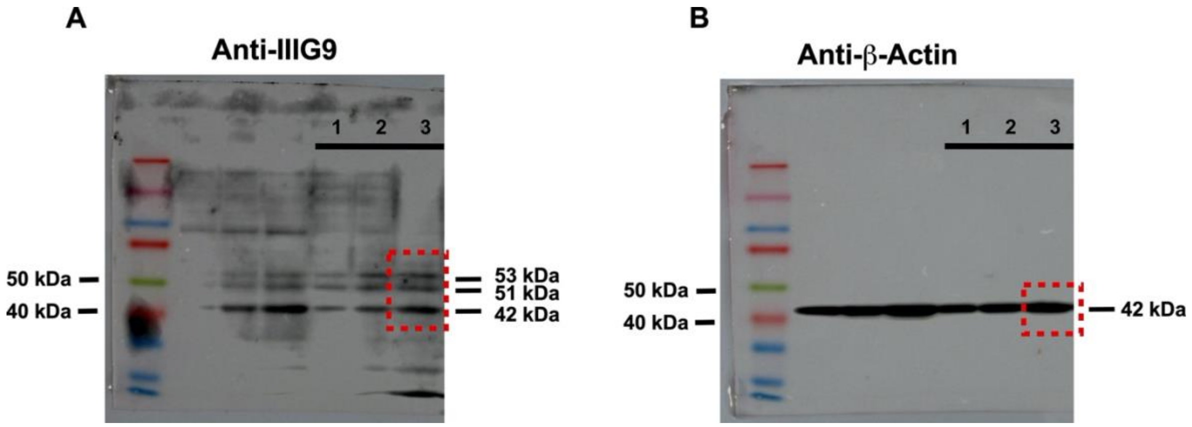

Suppl. Fig. 2. Unedited blot, figure 1b. Total protein extracts of adult ventricular wall (25 μg, 50 μg and 100 μg) were resolved in a 5%-15% SDS-PAGE gel. The membrane was incubated with anti-IIIG9 (1:10,000) (A) and reblotted to detect β-actin (1:10,000) (B). The red broken lines indicate dropped bands.

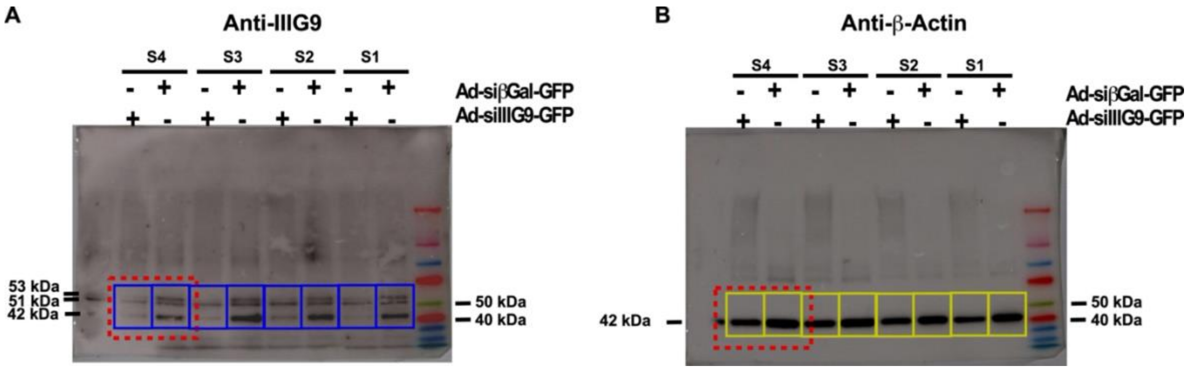

Suppl. Fig. 3. Unedited blot. Figure 1h. **A.** Total protein extracts of adult ventricular wall (10 μg) at 6 days post-transduction with Ad-siβGal-GFP or with Ad-siIIIG9-GFP were resolved in a 5%-15% SDS-PAGE gel. The membrane was reblotted to detect β-actin (B). Red broken lines indicate dropped bands. **C.** Bands used for densitometric analysis, blue boxes for IIIG9 and yellow boxes for β-actin.

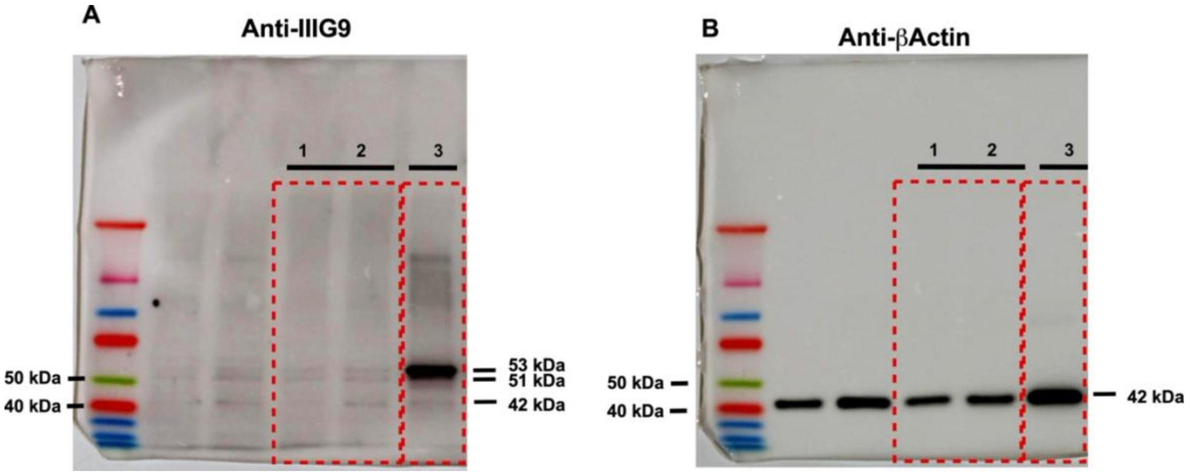

Suppl. Fig. 4. Western blot analysis for IIIG9 expression comparing the size of protein band detected in samples of trachea and lateral ventricular wall isolated from adult rats. (**A**) Total protein extracts of lateral ventricular wall (15 μg/lane) and trachea (10 μg and 20 μg) were resolved in a 5%-15% SDS-PAGE gel. (**B**) The membrane was incubated with anti-IIIG9 (1:10,000) and reblotted to detect β-actin (1:10,000).
